# Contextual modulation of social and endocrine correlates of fitness: insights from the life history of a sex changing fish

**DOI:** 10.3389/fnins.2015.00008

**Published:** 2015-02-03

**Authors:** Devaleena S. Pradhan, Tessa K. Solomon-Lane, Matthew S. Grober

**Affiliations:** ^1^Department of Biology, Georgia State UniversityAtlanta, GA, USA; ^2^Neuroscience Institute, Georgia State UniversityAtlanta, GA, USA

**Keywords:** androgen, cortisol, parenting, social status, reproduction

## Abstract

Steroid hormones are critical regulators of reproductive life history, and the steroid sensitive traits (morphology, behavior, physiology) associated with particular life history stages can have substantial fitness consequences for an organism. Hormones, behavior and fitness are reciprocally associated and can be used in an integrative fashion to understand how the environment impacts organismal function. To address the fitness component, we highlight the importance of using reliable proxies of reproductive success when studying proximate regulation of reproductive phenotypes. To understand the mechanisms by which the endocrine system regulates phenotype, we discuss the use of particular endocrine proxies and the need for appropriate functional interpretation of each. Lastly, in any experimental paradigm, the responses of animals vary based on the subtle differences in environmental and social context and this must also be considered. We explore these different levels of analyses by focusing on the fascinating life history transitions exhibited by the bi-directionally hermaphroditic fish, *Lythrypnus dalli*. Sex changing fish are excellent models for providing a deeper understanding of the fitness consequences associated with behavioral and endocrine variation. We close by proposing that local regulation of steroids is one potential mechanism that allows for the expression of novel phenotypes that can be characteristic of specific life history stages. A comparative species approach will facilitate progress in understanding the diversity of mechanisms underlying the contextual regulation of phenotypes and their associated fitness correlates.

## Introduction

Most organisms exhibit distinct developmental and reproductive stages during their life cycle. Physiological factors are critical orchestrators of life history transitions and coordinate dynamic, context-specific activities to increase fitness. In reproductively mature individuals, activities generally focus around nutrition and reproduction, and in turn, both of these activities are broadly dependent upon temporal patterns associated with circannual and seasonal rhythms (Ball et al., 2004; Prendergast et al., 2009). Within this realm, steroid hormones, the molecules of focus in this review, regulate phenotypes such as behavior and morphology in response to environmental change (Wingfield et al., 1990; Remage-Healey and Romero, 2001).

**Figure 1:**
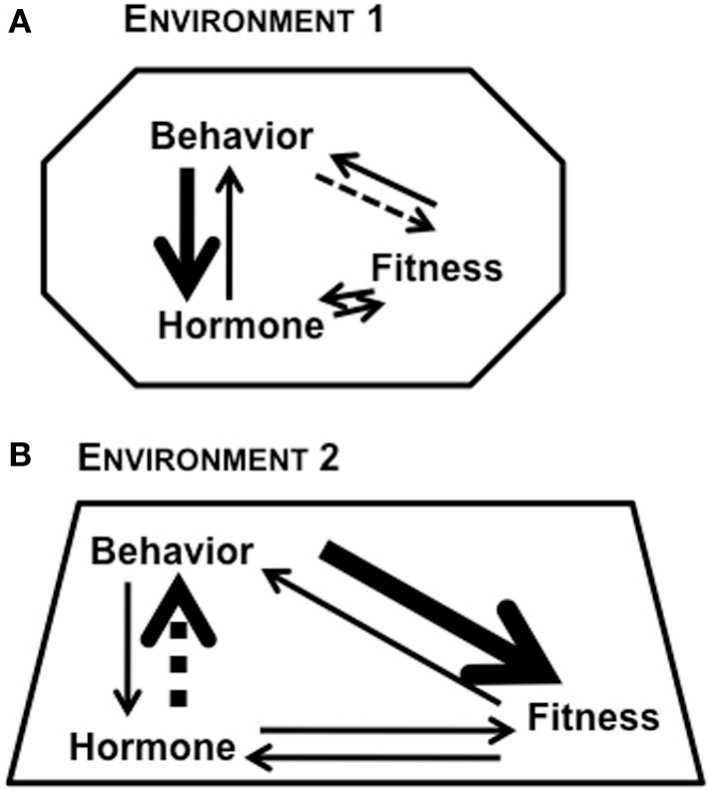
**Reciprocal relationships between hormone, behavior, and fitness of an individual**. The strength of relationship between any two factors can be modulated based on context and the environment in which the organism lives. The different shapes in **(A)** Environment 1 and **(B)** Environment 2 represent the role of context in modulating the bi-directional links between any two factors. The varying thickness of arrows represents the complexity of links between factors.

The environment can cause both predictable and unpredictable changes in endogenous hormones, either at the level of synthesis or signaling cascades to cause downstream consequences (Pradhan and Soma, 2012). Depending upon the particular environment, the behavior expressed by an individual (in isolation or toward conspecifics) can be modulated by steroid hormones, which has fitness consequences (Figure 1). The local environment can modulate the internal physiological state of an organism, with subsequent effects on both behavior and fitness, while the geographic location of the habitat can also serve as a selective pressure regulating physiological mechanisms (Nelson, 2011; Wingfield, 2012). For example, the patterns of genotype, phenotype, and plasticity of expression are rather different for animals living in the Tropics, Temperate, and Arctic regions (Wingfield et al., 1990; Borg, 1994; Wingfield and Hunt, 2002). In addition, whether data come from natural vs. laboratory environments has a major impact on their interpretation. For example, natural populations have high hormone levels, and natural predator stress can cause a greater glucocorticoid response than what can be simulated in a laboratory (e.g., via restraint) (Newman et al., 2013). It is imperative that sex steroids and glucocorticoids have differential responses to environmental stressors (Narayan et al., 2012) so that during a stressful event, the tradeoffs favor an instinct for survival, rather than reproduction. Given that fitness, hormones, and behavior are reciprocally associated, integrative studies that examine all three factors will provide critical insights into the evolution of life history strategies and regulation of processes that optimize fitness.

Sex changing fish are excellent models for understanding the fitness consequences associated with the behavioral and endocrine variation among individuals. In theory, an optimal life history strategy for any species involves regulating behavior, physiology, and reproduction to match each context an individual finds itself in, as context changes over time, in order to maximize fitness (Horn and Rubenstein, 1984). Sex change allows individuals to reproduce as a different sex depending on the context or environmental conditions and can provide major increases to lifetime reproductive success (Warner et al., 1975). Generally, sex change should be favored in species for which male and female reproductive success differs over a lifetime and the reproductive success of one sex increases more rapidly than the other with time, age, or size. For example, in protogynous species, individuals reproduce as females when younger, smaller, and lower in social status and then as males when older, larger, and higher in social status (Ghiselin, 1969; Warner, 1975; Munday et al., 2006; Godwin, 2009). Functional sex change requires that an individual reproduce successfully as one sex and then as another. Integrative studies on sex change investigate multiple levels of analyses, including (1) behavior, such as establishing dominance initially and reproduction (courtship, mating, and parenting) at a later stage; (2) morphology, such as color change or gonadal and external genital rearrangement; (3) physiology, such as changes in profiles of sex hormones in the gonad, systemic circulation, and brain; and (4) reproduction, such as presence of fertilized eggs (Reavis and Grober, 1999; Black et al., 2005b; Rodgers, 2007).

**Figure 2:**
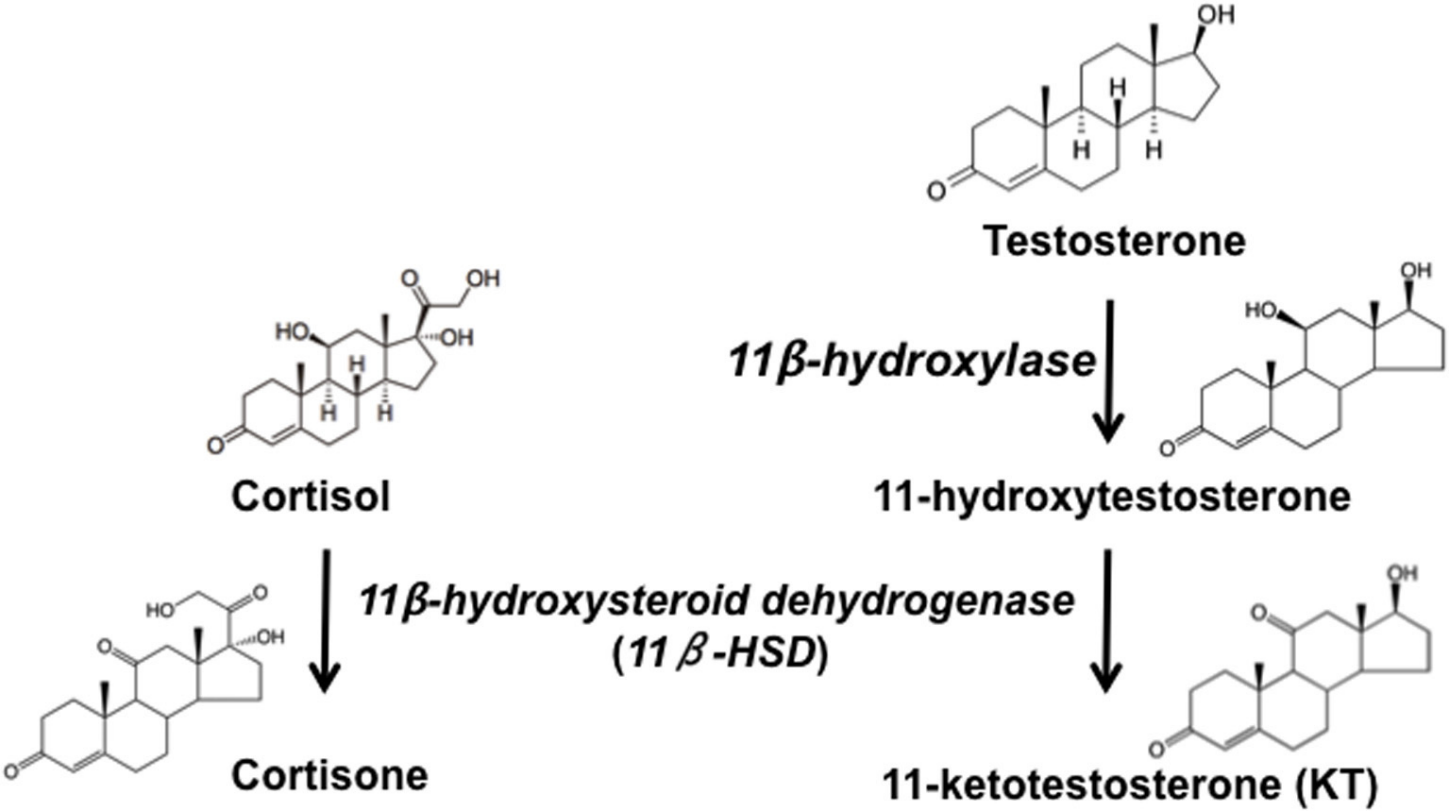
**Simplified pathway of steroidogenesis in fish**. Testosterone is converted to 11-Ketotestosterone (KT) via the sequential action of 11β-hydroxylase, which converts KT to 11β-hydroxytestosterone (11β-OHT), and 11β-hydroxysteroid dehydrogenase, which converts 11β-OHT to KT and cortisol to cortisone. Adapted from Pradhan et al. (2014c).

**Figure 3:**
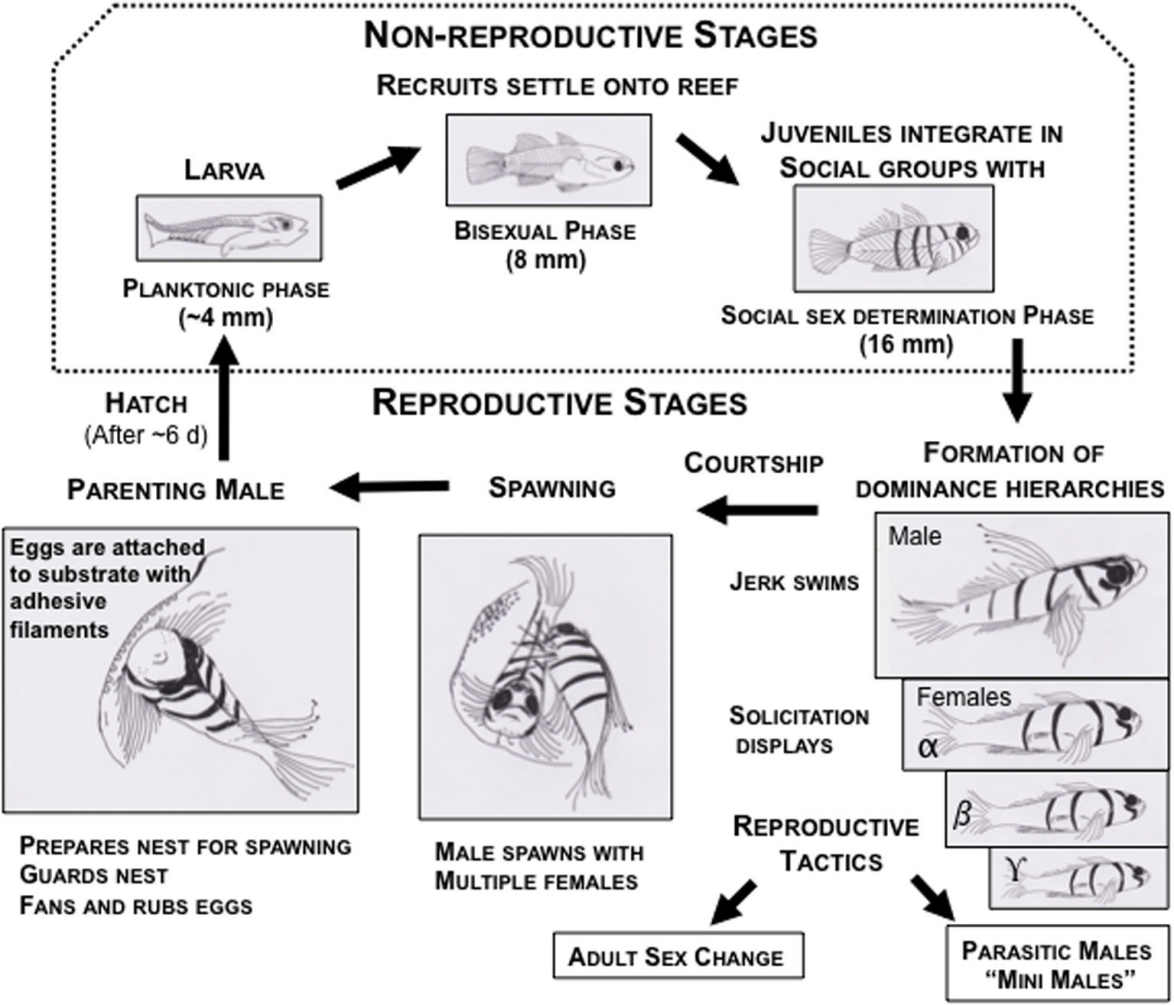
**Life cycle of bluebanded gobies, *Lythrypnus dalli*, depicting the life history stages during the breeding season in waters off the coast of Santa Catalina Island, California**. The dotted box encompasses the non-reproductive stages, which are not discussed in this review. The remainder are parts of the reproductive stages, and each of these comprise sub-stages of territorial aggression, dominance hierarchies, courtship (male jerks and female solicitation), spawning, and parenting. In the laboratory, social groups are easily set up under conditions that are permissive for natural sex change and for spawning. These fish also show alternative reproductive strategies to increase lifetime reproductive success, such as socially controlled bi-directional adult sex change and parasitic male morphs.

The goal of this review is to bridge the gaps in understanding the links among fitness, hormones, and behavior when animals are working to adjust these factors in a variety of ethologically or ecologically relevant environmental/external contexts. First, we describe the fitness component of our model and propose how we can improve our understanding of behavioral endocrinology by incorporating fitness measures in our experiments. Second, we outline how to improve our understanding of endocrine regulation by considering different levels of analyses within the endocrinological context. The steroid hormones discussed herein [testosterone (T), 11-ketotestosterone (KT), 17β-estradiol (E_2_), and cortisol (F)] are linked through steroidogenic conversion pathways (Figure 2) and can be produced and transduced at multiple sites across the body axis. Third, we discuss the integration of social and endocrine contexts, which allows us to better interpret the mechanisms that regulate transitions among phenotypes. Finally, we propose a model for how these factors interact to influence the expression of phenotype. Being cognizant of these different components and levels of analyses is especially important when designing experiments and interpreting results. Throughout this review, we present examples from several different vertebrate species but focus on data from the remarkable life history of the bi-directionally hermaphroditic, highly social marine fish, the bluebanded goby, *Lythrypnus dalli* (Figure 3).

## Fitness

To effectively incorporate a fitness component into our studies of hormones and behavior, it is critical to discuss how fitness is defined and measured. A variety of empirical measures of fitness have been utilized in field and laboratory experiments. Although the conceptual definition of fitness is widely agreed upon by scientists, a large number of operational definitions are presented in the literature. In fact, the significance and consequence of these different definitions has itself been the subject of study (Barker, 2009; Orr, 2009). In order to decide on the appropriate fitness component or proxy for a given experimental paradigm, a thorough analysis is necessary to identify constraints to measuring fitness. Through our synthesis, we aim to emphasize the value of integrating fitness measures into mechanistic studies of behavioral endocrinology.

### Broad definition

Generally, individual fitness is defined as passing genetic material to the next generation. However, fitness may also refer to a genotype, a population, or a species. Fitness is defined and measured differently at these levels of analyses and, as a result, requires the appropriate tools. For example, while the fitness of an individual may be reasonably estimated directly (i.e., by counting all the surviving offspring of an individual) or as its components, survival or reproductive success (e.g., offspring produced during an experimental time period), a population understanding of fitness might utilize genetic analyses of quantitative trait loci along with a variety mathematical fitness statistics (Barker, 2009; Orr, 2009). For the purposes of this article, which broadly concerns fitness, hormones, and behavior, we will focus on individual, i.e., phenotypic, fitness.

### Operational definition

Given the number of fitness definitions present in the scientific literature, it is critical for each study to provide a clear operational definition. From a survey of fitness definitions by Barker (2009), we would like to highlight three important considerations in defining fitness that are especially applicable to individual fitness. First, experimental quantification of fitness and selection pressures should distinguish between two phases of selection. Within a generation, fitness differences among individuals can lead to change in the frequency distribution of phenotypes (e.g., via differential survival rates). If the fitness differences among individuals have a genetic basis and, therefore, are heritable, then these fitness differences will also lead to a change in the frequency distribution of phenotypes and genotypes in the next generation (Barker, 2009). Second, definitions frequently vary in the number of generations considered. Based on one definition, an individual might qualify as having high fitness if a large number of offspring are produced. By another definition, those offspring might also be required to produce a large number of offspring. An even more stringent definition might require that genetic material be passed down across multiple generations (Ellis, 1995; Barker, 2009). As discussed in more detail below, experimental limitations often affect how many generations can feasibly be quantified. Third, the importance of context in quantifying fitness is stated explicitly in some definitions, but not in others. The fitness of individuals must be compared within the same environmental context, for example, because gene by environment interactions can significantly affect the fitness of a given phenotype (Greenfield and Rodriguez, 2004; Barker, 2009). It is also critical to consider the life history stage of the individuals when fitness is quantified (Orr, 2009). For example, fish that undergo adult sex change, a primary focus of this paper, produce dramatically different numbers of offspring as females than as males (Warner, 1975; Munday et al., 2006). Estimates of reproductive success early in life, therefore, would fail to incorporate the increase in reproductive success following sex change. Similarly, when quantifying fitness proxies (discussed below), such as fighting ability, there may be age- and life history-dependent differences in the success of fighting using different methods (Lailvaux et al., 2004); therefore, accurate estimates of ability require assessment at multiple life history stages.

### Measures of fitness

In studies of hormones and behavior, individual fitness is measured most often as its components or via the use of proxies. The primary components of fitness are survival and reproductive success. Individuals must survive into reproductive maturity in order to have non-zero fitness, and for species in which individuals reproduce more the longer they live, age may be directly related to reproductive success. African elephants in musth, for example, experience an increase paternity success as they age (Hollister-Smith et al., 2007). The relationship between survival and reproductive success is not simple in all species. In *L. dalli*, territorial males are larger and likely to be older than subordinate females (Behrents, 1983), but the association between age (e.g., survival) and reproductive success is not likely to be causative. For species that experience reproductive senescence (e.g., Angelier et al., 2006; Aubry et al., 2009), increased survival beyond a certain age may or may not contribute significantly to reproductive success and, therefore, fitness. Finally, there may be a tradeoff for some species (e.g., elephant seals) between dominance/high reproductive success and life expectancy (Ellis, 1995).

Reproductive success refers to the number of offspring produced by an individual, usually within a time period shorter than a lifetime (to distinguish from a direct measure of fitness). As discussed above, definitions vary with respect to the number of generations genetic material must be passed down (Barker, 2009). Although an ideal measure would involve tracking genes across infinite generations, a strong definition of reproductive success is the number of offspring that survive until reproductive maturity (Ellis, 1995). Impressively, researchers studying chacma baboons (Silk et al., 2009), collared flycatchers (Brommer et al., 2005), great reed warblers (Hasselquist, 1998), red-winged blackbirds (Forbes, 2011), feral horses (Cameron et al., 2009), dolphins (Frère et al., 2010), hyenas (Holekamp et al., 2012), meerkats (Hodge et al., 2008), and others (Ellis, 1995) all include reproductive success measures based on offspring survival and maturation. In practice, many reproductive proxies are used to estimate reproductive success, such as courtship (e.g., Shamble et al., 2009), mate choice (e.g., Clutton-Brock and McAuliffe, 2009), mating opportunities or attempts (e.g., Chen et al., 2011), successful copulations (e.g., White et al., 2010; Formica et al., 2011), the number of eggs laid (e.g., Ros et al., 2009), or the number of offspring born (e.g., Bell et al., 2011). While a number of reproductive proxies have been validated empirically by correlating the proxy with a more robust measure of reproductive success, others have not. In fact, some common proxies are not related at all to reproductive success in particular species, for example, male macaque copulation frequency (Ellis, 1995). Additionally, measuring different proxies within the same species can lead to different conclusions about the fitness consequences of a phenotype (Wong and Candolin, 2005). In the pygmy swordtail, although females prefer males with blue body coloration, gold-colored males outcompete blue males in agonistic interactions (Kingston et al., 2003). Dominance is itself a powerful predictor of status in many species (Ellis, 1995), and gold male dominance may constrain females' ability to act on their preference (Kingston et al., 2003).

Morphological and behavioral proxies are also common stand-ins for fitness and its components (survival, reproductive success). In some systems, the connection between morphological traits and fitness is well-documented. For example, male tail white in dark-eyed juncos has a genetic basis and is influenced by diet and age. Females prefer males with more tail white, males with more tail white tend to win male-male aggressive interactions, and larger males with more tail white produce offspring with more females (Hill et al., 1999; McGlothlin et al., 2005, 2007). Body parts of exaggerated size, such as weapons, horns, or claws, have also been linked to fighting ability, attractiveness, and reproductive success (Emlen, 2008). As with other proxies, however, rigorous tests of these associations are critical because, for example, empirical evidence linking ornamentation to body condition is generally lacking (Cotton et al., 2004), and the association between female ornamentation and offspring quality varies considerably (Nordeide et al., 2012). Perhaps the most common behavioral proxy for fitness is social dominance (Wilson, 1980; Ellis, 1995). Dominants may gain higher reproductive success by suppressing subordinate reproduction (Barrett et al., 1993; Clarke and Faulkes, 2001; White et al., 2002; Fitzpatrick et al., 2008), monopolizing mating opportunities, producing more offspring, and experiencing lower rates of abortion, egg loss, and offspring mortality (Smuts and Smuts, 1993; Trunzer et al., 1999; van Noordwijk and van Schaik, 1999; East and Hofer, 2001; Young et al., 2006; Robbins et al., 2007; Heg and Hamilton, 2008; Henry et al., 2013).

### Exercise caution when interpreting fitness data

Once a fitness proxy has been validated in the same or in a related species, morphological and behavioral traits are often used as stand-ins for fitness. Given that individual fitness is sensitive to context, however, it is important to differentiate between inferred and empirically quantified fitness consequences. While in many cases scientists have demonstrated consistent links, across species, between a given trait and fitness (Ellis, 1995; Emlen, 2008), it is critical not to assume fitness consequences or generalize without evidence across species and contexts. To demonstrate the importance of rigorously testing fitness hypotheses, we would like to highlight a few examples of unexpected fitness relationships. First, in the banded mongoose, there is a strong reproductive skew. Dominant females will evict pregnant subordinates from the social group, which causes abortion. Interestingly, dominant females that evict subordinates suffer reproductive costs, including lower offspring weight at birth and independence, as well as lower offspring survival (Bell et al., 2011). Second, in many hierarchical species, there is an association between status and glucocorticoid levels. Although subordinate status is often considered the more “stressful” social status, comparative data suggest that in approximately half of the species surveyed, dominant individuals were more stressed (Creel, 2001), which can itself be associated with negative fitness consequences (e.g., Sapolsky, 2005). Third, in many species, males adopt different reproductive strategies. In some species, there is evidence that alternative male morphs, long considered a less successful strategy than the more visible territorial or bourgeois male, may be equally reproductively successful (Taborsky, 1994). Finally, in a cooperatively breeding cichlid, subordinate females assist in caring for eggs produced by the dominant breeding pair. Neither kin selection nor the “pay-to-stay” hypothesis, which suggests group membership is the benefit to alloparenting, fully explains why subordinates help. Instead, subordinate females that alloparent are able to secure substrate on which to lay their own eggs (Heg et al., 2009). Ultimately, failing to rely on robust fitness measures to establish whether a trait, behavior, or phenotype is successful can lead to misleading or false statement about fitness consequences that may get repeated while remaining untested.

### Constraints of incorporating fitness measures

In discussions about fitness, it is also important to acknowledge the practical constraints that make direct quantifications of fitness unfeasible, impossible, or arguably irrelevant to the specific research question. Although we have made clear the benefits of measuring fitness or fitness components directly in order to make strong statements about individual/phenotypic success and selection, we can recognize Dobzhansky's dictum about the importance of evolution for understanding biological phenomena (Dobzhansky, 1973) without calling for all experiments to be directly concerned with fitness measures. Some common constraints to experimentally measuring fitness include species that do not reproduce in the laboratory yet provide important insight into biological questions. For species that do reproduce in the laboratory, neither parents nor offspring are exposed to ethologically-relevant social and environmental pressures; therefore, extrapolations about fitness could be difficult to justify. The life history of some species makes it difficult to empirically quantify reproduction, such as species that are slow to mature and reproduce rarely. Similarly, life history transitions affect the ability to quantify offspring characteristics like condition, survival, and subsequent reproductive success if, for example, species undergo pelagic or dispersal periods. In field studies, species may cover too much area to track effectively, and courtship and mating events may be purposefully cryptic or difficult to observe due to crepuscular or nocturnal timing. Tracking fitness across generations typically requires long-term studies, which can be practically and monetarily difficult to conduct. Finally, in mechanistic studies, manipulations of hormones or neuropeptides alter physiology and behavior over relatively short periods of time, during which effects on reproduction or survival may not be feasible or generalizable to what might occur over a lifetime.

### Integration of behavioral endocrinology and fitness

Hormones, behavior, and fitness are reciprocally related (Figure 1), and incorporating fitness measures may further elucidate the links between hormones and behavior, which are commonly measured together. In behavioral endocrinology, an important question to ask is whether hormones affect fitness indirectly via an affect on behavior or whether the fitness effect is direct. It must be clarified, however, that direct and indirect effects of hormones on fitness are not mutually exclusive. These effects can be important during different phases of life history of the same individual, and in many cases, it is challenging to tease apart direct and indirect effects. Direct effects of fitness on reproductive success could occur via effects on reproductive biology and behavior, while an indirect effect on reproductive success might affect a non-reproductive behavior that consequently affects reproductive success (e.g., social behavior).

Although the underlying mechanisms are far from simple, we will present some brief examples of direct and indirect fitness effects of two classes of hormones: glucocorticoids and androgens. Reproductive behavior can be energetically costly in terms of courtship displays (Fusani et al., 2014), spawning/mating (Watson et al., 1998), and parenting (Pradhan et al., 2014c, in press). For example, nesting male peacock blennies judge whether they can accept another clutch of eggs based on their energetic state (Olsson et al., 2009). Glucocorticoids are directly involved in the regulation of energy and behaviors related to acquiring energy, such as foraging (Schneider and Wade, 1999); therefore, glucocorticoids can directly affect the expression of reproductive behaviors. Glucocorticoids are also directly affect reproductive biology. The glucocorticoid cortisol, for example, is necessary for vitellogenesis (Brooks et al., 1997). Androgens also have direct effects on reproductive success, for example, via effects on spermatogenesis (Walker, 2011), expression of secondary sexual characteristics (Saraiva et al., 2010) and/or via androgen-dependent reproductive behaviors such as courtship displays (Vasconcelos et al., 2012; Schlinger et al., 2013) and parenting (Trainor and Marler, 2001; Foerster and Kempenaers, 2005). With respect to indirect effects, both glucocorticoids and androgens are associated with social status and agonistic behavior in a number of hierarchical species. High status individuals typically have a reproductive advantage over subordinates (Ellis, 1995), thus, behaviors such as territorial defense, dominance/submissive behavior, and aggression can all indirectly affect reproductive success (Foerster and Kempenaers, 2005). Both glucocorticoids and androgens are involved in numerous biological processes, and chronic exposure could impact fitness via more than one mechanism to incur reproductive costs (Wingfield et al., 2001; Breuner et al., 2008).

Sex changing fish are an excellent model for understanding how fitness can enhance our understanding of the causes and consequences of behavioral and endocrine state. Sex changing fish are useful because under a variety of contexts, hormones, behavior, and fitness can all be feasibly quantified. Furthermore, the foundational cannon of research on sex changing fish focused on fitness. As a life history strategy, sex change allows an individual to maximize reproductive success (Ghiselin, 1969; Warner, 1975). For example, for territorial species, young and small fish are not competitive territory holders. If males are the territory holders, an effective reproductive strategy would be to reproduce as female when young and small and then as a male when older, larger, and able to defend a territory. In the following section, we will discuss how the contextual regulation of fitness, hormones, and behavior has been measured in *L. dalli*.

### Insights from *Lythrypnus dalli*

A number of fitness proxies have been utilized in research with *L. dalli*, most of which approximate reproductive success. These include estimates of gamete production, gonad morphology, social status, and various reproductive measures of eggs in the nest. Extensive work has been done classifying sexual allocation in males, females (St. Mary, 1993), and alternative male morphs, mini males (Figure 3) (Drilling and Grober, 2005). Mini males are considered to be a parasitic male morph in *L. dalli*, based on physiological and morphological traits, although their behavioral strategies remain to be described (Pradhan et al., 2014b). Adult male and female gonads contain both types of gametes, but the investment is heavily skewed toward the sex that the individual behaves and reproduces as St. Mary (1993). Mini males have an important allocation difference compared to nesting males. Mini males utilize the accessory gonadal structure to store sperm, while nesting males store mucus (Drilling and Grober, 2005).

**Figure 4:**
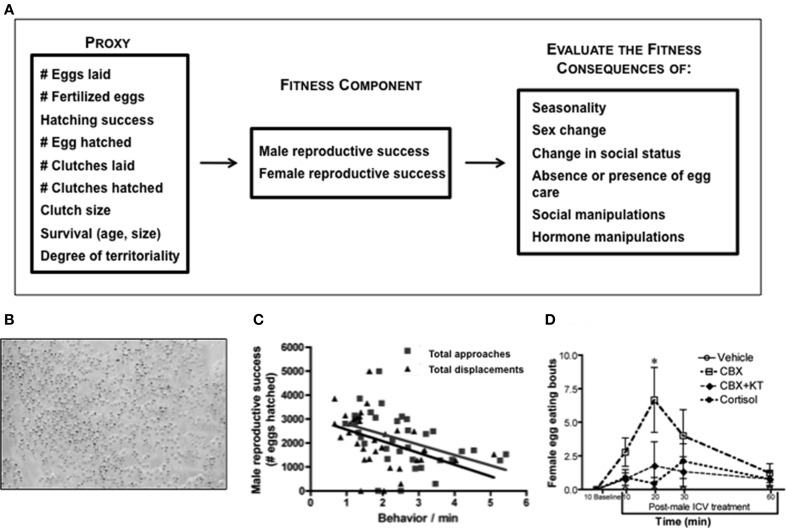
**Integration of fitness and behavioral neuroendocrinology in *Lythrypnus dalli*. (A)** Examples of fitness proxies that are commonly used to estimate reproductive success. One or more of these measures can be evaluated during different life history stages and within a particular endocrinological or social context. **(B)** Eggs at different stages of development, such as newly laid and eyed. **(C)** In stable social groups male reproductive success is negatively associated with the frequency of approaches and displacements in the social group (total approaches is male, alpha, beta, gamma approaches). Adapted from Solomon-Lane et al. (2014). **(D)** Intracerebroventricular (ICV) treatment of males presented females with a new social opportunity, permitting them to enter the nest and eat eggs. CBX, Carbenoxolone; KT, 11-ketotestosterone, ^*^p < 0.05. Adapted from Pradhan et al. (2014c).

Social groups of *L. dalli* are composed of a dominant male and multiple subordinate females. More than one female (and usually all females) lays eggs in the nest of the male, resulting in male reproductive success that is multiple times higher than any individual female in the group (Behrents, 1983). Female *L. dalli* routinely lay eggs and males readily parent in the laboratory (Pradhan et al., in press). Using sequential digital images of eggs in the nest, we can quantify the number of eggs laid, the number of clutches laid, average clutch size, inter-clutch interval, hatching success, and the number of eggs that hatch (Figure 4). Larval *L. dalli* are planktonic (Figure 3), making offspring survival and reproduction difficult to quantify in the laboratory and unfeasible in the field. Therefore, the number of eggs that hatch is our best estimate of reproductive success (Solomon-Lane et al., 2014).

In stable social groups, the quantity of eggs fertilized by the male is associated with the pattern of agonistic interaction in the group, especially among females (Solomon-Lane et al., 2014). More dominant females interrupt courtship solicitation displays by subordinate females and assume solicitation displays themselves (Pradhan et al., in press). Even though dominant and subordinate females display courtship at similar rates, number of eggs and number of eggs that advance to the “eyed” stage are positively associated with rates of dominant female courtship (Pradhan et al., in press). Therefore, agonistic interactions and social status could be used as one type of behavioral proxy. Once eggs are fertilized, males provide sole parental care, and they vary in their hatching success (Solomon-Lane et al., unpublished data), a quantitative measure of parenting efficacy, and in their rates of parenting (Pradhan et al., in press). In stable social groups, male reproductive success is negatively associated with the frequency of agonistic behavior, approaches and displacements, in the social group (Figure 4C) (Solomon-Lane et al., 2014). Males that fail to parent also suffer a reproductive cost because female *L. dalli* cannibalize eggs in an unguarded nest (Figure 4D) (Pradhan et al., 2014c). Following the removal of a male from the social group, the dominant female changes sex to male, and functional sex change is typically evaluated based on the ability of the new male to fertilize eggs (Reavis and Grober, 1999; Rodgers, 2007). Successful sex change is a life history transition that dramatically increases reproductive success (Behrents, 1983), and because this species functions as a sequential protogynous hermaphrodite, maleness itself (indicated by behavior, genital papilla morphology, and gonadal sex allocation) is a proxy for both survival and reproductive success.

## Endocrine context

Steroid hormones respond to environmental signals in order to integrate environmental information into behavioral command decisions (Alcock, 2001). These signals can be detected only when the endogenous state of the organism is primed, via receptor expression, to respond. Signal transduction occurs via cellular and molecular mechanisms and must be considered within the context of response location (e.g., anatomical site). The end goal of the signal is to induce a phenotypic effect (Ball and Balthazart, 2008). Based on the organism studied, there are several different types of biological samples that could serve as proxies of steroid bioavailability. To understand the mechanisms by which steroids regulate structure and function, a variety of approaches to both quantifying steroids and manipulating steroid availability have been developed. This work has elucidated the multiple levels of endocrine context that should be considered when assessing the variation in behavior and fitness across life history transitions. We will now summarize the proxies of steroid function that are commonly used, how mechanisms of steroid function are investigated via endocrine manipulations, and the associated limitations.

### Proxies of steroid function

Collectively, steroids affect many facets of phenotype, and specific steroids can have multiple effects (Nelson, 2011). These steroid functions include, but are not limited to, production of gametes, maintenance of homeostasis, activities related to survival and reproduction, development of secondary sexual morphological characteristics, and various aspects of social behavior (Wingfield et al., 1990; Borg, 1994; Cardwell et al., 1996; Trainor and Marler, 2001). At a fundamental level, the potent physiological impacts of steroid hormones occur primarily via steroid binding to membrane or nuclear associated receptors that up-regulate specific biochemical pathways via intracellular mechanisms, which then affect protein translation. As this level of experimental analysis is not possible for most behavioral investigations, proxies of steroid action are most often relied upon.

**Figure 5:**
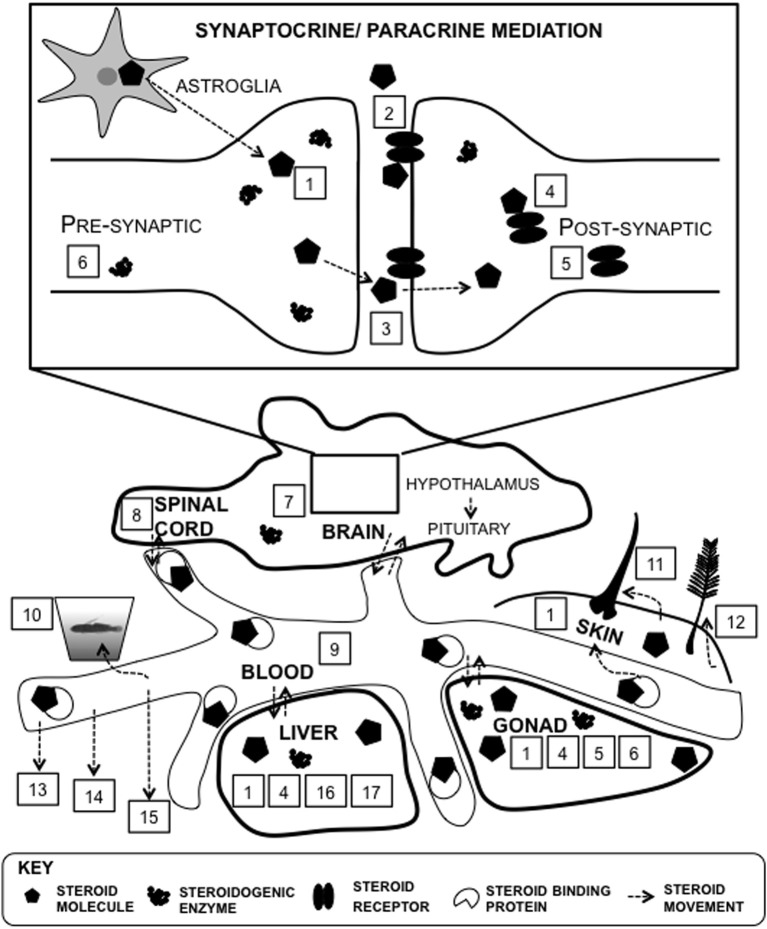
**Proxies of steroid function at multiple levels of analysis**. There are many different approaches to determine steroid levels in organisms. Steroids can be measured within particular tissues such as the brain, and in systemic circulation, such as plasma. Direct proxies within brain tissue include steroid signaling molecules such as receptors and steroidogenic machinery, such as enzymes (#1–8): 1, tissue; 2, membrane receptor protein; 3, synapse; 4, intracellular receptor protein; 5, steroid receptor mRNA expression; 6, steroidogenic enzyme mRNA expression; 7, steroidogenesis; 8, cerebrospinal fluid. Steroids can be synthesized in astroglia and within the the synaptic bouton (Saldanha et al., 2011). There are many proxies of systemic measures (#9–17): 9, plasma; 10, water-borne; 11, hair/fur; 12, feather; 13, saliva; 14, urine; 15, feces; 16; steroid metabolism; 17, steroid conjugation.

Which proxy is the most accurate representation of steroid levels that trigger and maintain activational effects in an organism? The answer to this question is anything but clear-cut because all proxies are indirect measures and have limitations (see below). There are many different levels of endocrine analyses, ranging from direct hormone measurements to receptor densities. Additionally, steroid receptor co-activators can modulate the downstream cellular response (Charlier et al., 2006). “Biomarkers” are naturally occurring molecules used to assess endogenous steroidal metabolites present in a biological sample of cells, tissues, and biofluids (Kotłowska, 2012). Often, the type of biological sample that is used as a proxy for steroid action is not based on accuracy, but rather on feasibility of collection and steroid detection (Figure 5). This results in many different levels of endocrine analyses, ranging from direct hormone measurements to receptor expression and binding kinetics. The site of steroid action—the cell—is probably the most appropriate location to measure levels of active steroids, but few techniques allow for the determination of cellular steroid levels associated with the production of specific behaviors. Microdialysis is a technique that allows for the determination of steroids in the extracellular fluid within specific regions of the forebrain of live, behaving animals (Ikeda et al., 2014; Rensel et al., 2014). While this may not be equivalent to steroid levels within specific cells, microdialysis is currently as close as we can get to measuring brain steroid levels in awake, behaving animals. In addition to sequestering steroids produced in the periphery, the brain is a remarkably heterogeneous organ that has specific sites of steroidogenic enzyme expression, steroidogenesis, and sex steroid receptor expression (Carere et al., 2007; Schmidt et al., 2008; Do Rego et al., 2009; Arterbery et al., 2010; Pradhan et al., 2010b) Another valid proxy is the steroidogenic potential of the brain, which can be measured at specific regions or subcellular compartments using *in vitro* assays (Black et al., 2005a; Pradhan et al., 2010a,b). The evidence for synaptocrine signaling, which encompasses steroid synthesis within the presynaptic bouton and release in the synaptic cleft for rapid neuromodulation, has spurred a re-evaluation of the most widely used proxies of hormone measurements (e.g., plasma) (Peterson et al., 2005; Saldanha et al., 2011). The primary advantage of local synthesis within a traditionally “target” organ is the speed and localization of steroid action due to temporal and spatial specificity (Schmidt and Soma, 2008; Saldanha et al., 2011). Another advantage is the reduction in costs of steroid synthesis: less hormone would need to be produced locally in the brain relative to the quantities needed to induce system-wide (including the brain) elevations in steroids (Wingfield et al., 2001).

Technological advancements have allowed for assay and equipment development that maximizes steroid extraction, separation, recovery, detection, and sensitivity from a wide array of sample types (Makin et al., 2010; Taves et al., 2011). At a very crude level, the weight of gland or the tissue that synthesizes the hormone can be measured to estimate the possible steroidogenic output (Buhimschi et al., 2008; Weathington et al., 2012). This is an indirect measure of steroid-releasing potential of that tissue. The most common estimate of steroid levels is also an indirect measure: plasma or serum is widely used because it is easily collected from most model organisms. This measure represents the systemic levels in circulation and is based on the view that steroids produced in specific organs are free to permeate the general circulation. Direct measures of steroids within specific tissues (both glands and targets) have been used successfully in fishes (Genova et al., 2012; Lorenzi et al., 2012) birds (Schmidt et al., 2009; Charlier et al., 2010), and rodents (Corpéchot et al., 1981) and provide an understanding of steroid function at the site of action. Steroid load can also be estimated by measuring specific proteins because steroids are generally bound to carrier proteins, such as sex hormone binding globulins (Heinlein and Chang, 2002), corticosteroid-binding globulin, or albumin (Huddleston et al., 2007), for transport to target organs.

A variety of non-invasive sampling methods are necessary for diagnostic testing of disease, detecting contamination, assessing responses to an environmental stressor or endogenous physiological factor, understanding the mechanism of steroid action, detecting steroids of abuse (Heitzman, 1976; De Jager et al., 2011; Divari et al., 2011), and assessing the reproductive status of wild and livestock populations. Fecal samples are used in wild or threatened populations (Borque et al., 2011). Urine samples can be easily collected from cattle (Doué et al., 2012) and amphibians (Narayan et al., 2012). For small fish, another non-invasive procedure involves measuring steroids exuded into water via gills, osmotic exchange through skin, and excretion (Sebire et al., 2007). For this procedure, the animal is temporarily kept in a beaker containing clean water for a long enough period of time for steroids to accumulate. As is the case with many non-invasive techniques, demonstrating correspondence between plasma hormone levels and water borne hormone is straightforward and necessary (Gabor and Contreras, 2012). It should not be assumed that multiple proxies of steroid function co-vary. For humans, salivary hormones are often measured because of the easy, non-invasive nature of sample collection (Hansen et al., 2008). Other non-invasive proxies of steroid measurement include feathers (Koren et al., 2012), hair [grizzly bears (Macbeth et al., 2010); humans (Dettenborn et al., 2012)], yolk [reptiles (Huang et al., 2013); fish (Feist et al., 1990), birds (Sockman et al., 2006)], and cerebrospinal fluid (Heidbrink et al., 2010). Taken together, other than direct measures of steroids at the site of action, all other measures should be regarded as proxies of steroid function and it is not clear if any one of those proxies (e.g., plasma/serum) is a “better” measure of steroid function than less traditionally employed measures. Our view is that each system has unique constraints on what can be sampled and each sampling method comes with specific limitations with regard to interpretation.

#### Limitations of endocrine proxies

In both field and laboratory settings, there are many constraints to measuring steroids from various species, including rodents. When studying free-living species, natural history, seasonality, type of sample availability, and ease of sample collection are all important considerations. The specific endocrine proxy used is also contingent upon other factors such as whether the species is dangerous to interact with, ease or ability to trap animals, lifespan, age, and population size. The degree of invasiveness of a procedure and how often samples are collected are also important considerations. By studying a species in a laboratory, some of these limitations can be circumvented and confounding factors can be controlled. Even under laboratory conditions, however, we need to be aware of the limitations and consequences of different sampling methods. The site from which a sample is collected should be reflective of the experimental question of interest, and carefully interpreted as such. This is especially critical when comparing steroid levels across a variety of sample types (e.g., plasma, tissue, waste). Due to the heterogeneity of steroid distribution in blood (Taves et al., 2010), one must exercise caution in interpreting plasma levels because it contains concentrated steroids compared to whole blood or red blood cells and might be an overestimation (Hiramatsu and Nisula, 1987; Taves et al., 2010). Environmental stressors can also have differential effects on steroids and their binding to proteins (Taves et al., 2010). In addition, the part of the body that blood is collected from must be considered. For example, cardiac, caudal, and brachial plasma represent systemic steroid levels. In comparison, the jugular vein represents blood that is exiting the brain, and is, therefore, an indirect measure of brain steroids (Schlinger and Arnold, 1993; Saldanha and Schlinger, 1997; Newman et al., 2008). Factors such as half-life (Cavaco et al., 1997), rate of elimination (Leshchenko et al., 2006), and rate of conversion to other active or inactive steroids can vary (Schlinger et al., 2008) depending on the site of blood collection, further complicating the interpretation of blood based steroid measures (Schmidt et al., 2008). Salivary samples are often collected from humans; however, context-specific variation limits its ability to be interpreted (Hansen et al., 2008; Kudielka et al., 2009).

### Endocrine manipulations

Manipulative mechanistic studies have been integral to our understanding of the regulation of behavioral and morphological phenotypes. Steroids can be manipulated locally, in particular tissues of interest, or systemically. The context of the endocrine manipulation (e.g., anatomical location or form of manipulation) is critical to consider relative to the particular questions of interest. Classically, steroids have been administered chronically, founded on the theory that steroids take hours or days to exert their phenotypic effects via classical genomic mechanisms. However, some effects of steroid hormones occur on a much shorter time-scale, over seconds or minutes, via non-genomic mechanisms. Hence the appropriate type of manipulation and route of administration must be considered. Again, any steroidal manipulation should be performed within well-defined environmental contexts that are relevant to the life history of the animal being studied. We will now summarize some types of endocrine manipulations and discuss the associated limitations.

#### Systemic manipulations

Traditionally, studies on reproductive endocrinology focus separately on gonadally produced sex steroids and adrenally (or interrenally in fish) produced glucocorticoids. Early in the development of behavioral endocrinology, extirpation and replacement led to the discovery of blood borne “factors” that were released into circulation from the testes and controlled phenotype. In 1849, Arnold Berthold conducted a series of seminal experiments in which he either removed and/or transplanted testes in young male chickens (Quiring, 1944). He found, that removing the testes prevented the development of secondary sexual characteristics and the expression of male-typical sexual and aggressive behaviors, while transplanting testes from other birds rescued these traits (Quiring, 1944). Terminal examination of these animals in adulthood led him to conclude that substances released by the testes into blood are transported throughout the body, and largely to the nervous system (Quiring, 1944). Accordingly, systemic manipulations that investigate regulatory effects of hormones have largely focused on manipulating peripheral tissues by removal of source of the hormone (gonadectomy or adrenalectomy). There are several other methods by which peripheral hormones can be manipulated. Pharmacological manipulations that inhibit steroidogenic enzymes or serve as receptor antagonists can prevent the downstream effects of steroids (Nelson, 2011). After demonstrating the loss of phenotype in response to steroid removal, subsequent delivery of the hormone(s) under investigation can rescue or restore normal functioning. This step is critical for identifying the mechanism of specific steroid action. Intraperitoneal (IP) manipulations, such as injections and implants, are most often used to replace steroids after gland removal. Hormone implants (IP or subcutaneous, Fuenzalida, 1950) can be of several types and include incorporation into beeswax (Pradhan et al., 2014c), pure crystalline pellets (Pradhan et al., 2014a), controlled released pellets (Fuenzalida, 1950), *in situ* forming microparticle implants (Castillo-Briceno et al., 2013), and silastic tubing implants (Damassa et al., 1977). Additionally, steroids can also be ingested via mixing in food (Remage-Healey and Bass, 2006). Finally, on amphibians, non-invasive methods of steroid delivery have included patch-delivery (Knapp and Moore, 1997) or direct application on bare skin by dissolving in a vehicle (Belliure et al., 2004).

#### Local manipulations

Local steroid manipulations are often insightful because they occur at the site of hormone action. The effects of local manipulations are also less prone to interference from any indirect effects of the hormones acting on other target tissues (Friedman et al., 1964). This can be accomplished by performing implants directly within the tissue in question, for example, in thymus (Friedman et al., 1964) and brain (Ramirez et al., 1964; Hartmann et al., 1966). For central hormone manipulations, cannulae have been used for long-term experiments (Huddleston et al., 2006) and intracerebroventricular (ICV) injections have been used for short-term studies (Tehranipour and Moghimi, 2010; Solomon-Lane and Grober, 2012).

#### Limitations of endocrine manipulations

Any type of *in vivo* experimental manipulation has limitations. Both peripheral and local manipulation can affect an organism in unintended ways, based in part on the degree of invasiveness of the procedure. Administration of hormones can produce a high degree of individual variation in the amount of systemic and local hormone levels (Pradhan et al., 2014a). Steroid treatment can have effects at many different biological levels and differential rates of endogenous feedback loops or breakdown might be involved (Pradhan et al., 2014a). Some hormones are extremely potent with activating effects occurring at low levels. The effects of hormones do not tend to increase with added hormone, demonstrating that steroids do not generally exert effects in a dose-dependent manner (Nelson, 2011). If hormone is administered after gonadectomy, then timing becomes an important consideration because much higher does of hormones are required for restoring behavior relative to maintaining it (Damassa et al., 1977). High doses can also have costly physiological side-effects (Wingfield et al., 2001). In addition, frequent delivery, such as injections, might be too invasive and induce a variety of unwanted endocrine side affects (Wallis et al., 2014). Some drugs do not penetrate the blood brain barrier (Leshchenko et al., 2006) and cannot be used to investigate central effects on behavior (Pradhan et al., 2014c). We can also use different manipulations to consider endocrine vs. paracrine functions of hormones and discriminate between systemic vs. local effects. Different steroids also have differential rates of release, binding, breakdown, conversion, sequestration, and lipophilicity (Babuska and Pyaka, 2006). Hence, once introduced into the body, the timescale of effects and mechanisms of action may differ based on the particular steroid structure. Another consequence of hormone delivery is that the manipulation of one hormone, for example using an enzyme inhibitor, can cause unintended affects on other hormones in the steroidogenic pathway. Steroidogenic enzymes often act in more than one direction, and the same enzyme can participate in the conversion of multiple steroids. For example, administration of carbenoxolone (CBX), an 11β-hydroxysteroid dehydrogenase (11β-HSD) inhibitor, increases F while decreasing KT in fish (see Figure 2) (Pradhan et al., 2014c). Carbenoxolone, a glycyrrhetinic acid derivative, inhibits the gastric enzyme, pepsin. It has been used to treat ulcers since the 1960s (Henman, 1970). While at least two isoforms of 11β-HSD have been described (Payne and Hales, 2004), and both are inhibited by CBX (Jellinck et al., 1993; Ge et al., 1997; Ma et al., 2011), fish have only one isoform (Arterbery et al., 2010). The phenotypic effects of CBX treatment in fish could be due to the effects of either KT and/or F. Thus, presence of multiple isoforms of an enzyme and the ability of a chemical to affect more than one enzyme are important considerations for understanding the effects of any pharmacological manipulation, central or peripheral. Additionally, measures of systemic hormone levels following peripheral manipulations are not indicative of the degree to which exogenous steroids remain elevated in specific tissues and thus affect their subsequent biological effects. For example, systemic hormone manipulation differentially impacts the levels of that hormone found in different tissues (Pradhan et al., 2014a). It is likely that elevated peripheral hormones are transported to tissue via the blood supply; however, the vascular supply to the brain may not have a significant impact on total brain steroid levels (Taves et al., 2010). Some of these limitations of peripheral delivery can be circumvented via local steroid manipulations.

### Insights from *Lythrypnus dalli*

**Figure 6:**
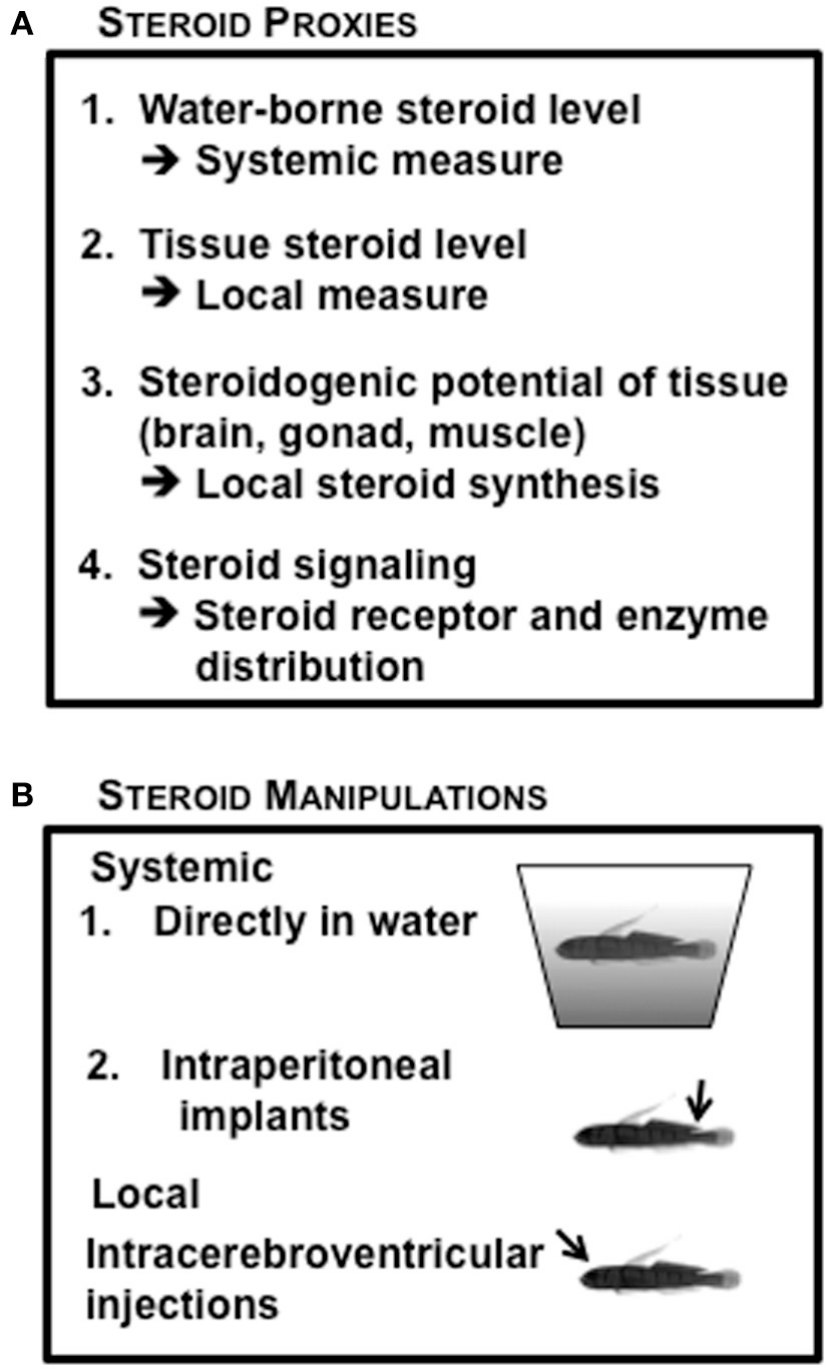
**Endocrine context of *Lythrypnus dalli*, commonly used in the laboratory. (A)** Steroid measurement proxies **(B)** Steroid manipulation approaches.

Given the substantial independent data indicating the importance of both systemic and local steroidogenic regulation, we have utilized a variety of steroid measurements in our studies of hormonal regulation of reproductive life history in the bluebanded goby (Figure 6A), including tissue steroid levels (Lorenzi et al., 2012); steroids released in water (Lorenzi et al., 2008), distribution of steroid receptors and steroidogenic enzymes in tissue sections (Schuppe et al., unpublished results), and the potential for steroid synthesis in particular tissues (Pradhan et al., 2014c). The endocrine manipulations we have used in *L. dalli* include systemic treatment using IP implants (Pradhan et al., 2014a), addition of steroids or steroid inhibitors to the water (Schuppe et al., unpublished results) and local brain manipulations using ICV injections (Solomon-Lane and Grober, 2012) (Figure 6B). This combination of endocrine measures with endocrine manipulations at different levels of analysis has allowed us to empirically question the traditionally accepted phenomenon that sex steroids are synthesized in gonads and transported by the blood to target organs. This is particularly relevant when viewing the brain as a target organ because a behavioral change does not always correspond with changes in circulating steroid levels (Pradhan et al., 2010b) or gonad specific changes in steroid levels (Pradhan et al., 2014c). If the traditional dogma that the gonad being the primary site of sex steroid synthesis is true, then systemic levels should closely reflect gonadal steroid production or levels of hormone extracted from gonadal tissue. This relationship should hold true for other target tissues as well; however, this does not appear to be the case (Pradhan et al., 2014b).

In stable groups of *L. dalli*, there are no status or sex differences in water-borne steroid levels (Solomon-Lane et al., unpublished results; Lorenzi et al., 2008). However, three different tissues, brain, gonads, and muscle, show different patterns of steroid levels among males and females of different statuses (Pradhan et al., in review; Lorenzi et al., 2012). For example, the brain has 3× higher androgen levels than the gonad (Lorenzi et al., 2012), subordinate females have higher brain T, KT, and F compared to dominant females (Pradhan et al., in review), females have several fold higher ovarian E_2_ compared to males (Lorenzi et al., 2012), and in mini males, brain and reproductive tissue levels of T, E_2_, and KT are higher than in the muscle (Pradhan et al., 2014b). When the dominant male is removed from the social group, all the females in the hierarchy show tissue-specific changes in hormone levels (Lorenzi et al., 2012). This independent regulation is important because the brain modulates behavior and must respond to rapid changes in social context. Other tissues, such as the gonad, often have more delayed responses (Lorenzi et al., 2012). The speed of the response may not be as critical for non-behavioral traits. To evaluate the effect of exogenous steroid hormones on steroid load within tissue, we IP implanted females undergoing sex change with KT. We found that even though the KT load increases markedly in brain, gonads, and muscles within only 5 days of treatment, elevations in KT levels varries across the three tissues (Pradhan et al., 2014a). This indicates that there is differential penetration, sequestration, or breakdown of KT in tissue. In addition, there is a negative relationship between the amount of KT absorbed from the pellet and the level of KT measured from the gonads of KT implanted fish, but not control fish. Thus, a systemic steroid manipulation affects local steroid loads quite differently.

When we consider the parenting stage of the life history, males that actively tend eggs have higher water-borne KT compared to experienced males not actively nesting, new males tending eggs, and females (Rodgers et al., 2006). To evaluate the role of KT in regulating male parenting, we performed two types of manipulations of 11β-HSD, an enzyme that synthesizes KT in both male gonad and brain (Pradhan et al., 2014c). First, using long-term IP implants of CBX, we found that KT synthesis is only transiently inhibited (Pradhan et al., 2014c). Second, using short-term ICV manipulation of parenting males, we show that the ratio of KT to F is rapidly (within 60 min) reduced in both brain and gonad (Pradhan et al., 2014c). Although water-borne KT is reduced by CBX in a dose dependent manner, F is not affected (Pradhan et al., unpublished results). Taken together, these results have important implications. First, local endocrine manipulations can have differential local and systemic effects. Second, endogenous steroids are linked through common enzymatic pathways, and manipulation of one enzyme can have differential and opposite effects on two active steroids.

## Social context

Interactions among conspecifics are frequent in social species and adaptive in particular environmental contexts (Wallen and Schneider, 1999). In the wild, an organism might experience variation in the type of social interaction with conspecifics and heteropecifics throughout its lifetime. For example, different types of interactions can emerge when dealing with parents, siblings, territorial or mate competitors, predators, prospective mates, and offspring. Two dramatic and highly escalated states of social interaction, sex and aggression, typically occur only under specific circumstances or contexts over the course of an organism's life history (Huber and Kravitz, 2010). Often, more than one type of interaction can overlap temporally, for example, warding off predators while providing brood care. Another change in social context can occur if a group member dies, and other individuals in a group compete to assume the open position in the hierarchy. For example, in *L dalli*, absence of the male from a social group creates a social context that is permissive for the highest ranking (alpha) female to establish dominance over the group, take over the nesting territory, and change sex to male (Pradhan et al., 2014a). Social context is also dynamic and is largely based on reciprocated interactions between or among individuals. For example, in a dyadic aggressive interaction, at least three components could be measured: (1) quality/intensity could range from threat displays to physical contact; (2) quantity could comprise the number of attacks and retaliations, and (3) duration of interaction would determine the timescale of interaction (Nilsen et al., 2004). In a status contest, individual variation in behavioral phenotype can lead to variation in the amount of time it takes for a resolution (Pradhan et al., 2014a). In addition, familiarity with a territory or residency status might also provide an advantage in social contests (e.g., “home advantage,” Fuxjager et al., 2009). In any experimental paradigm, the response of animals might differ based on the particular social context; therefore, subtle changes in social context should be taken into consideration.

### Integration of behavioral and endocrine context

Steroids can regulate social interactions in a context-dependent manner. To control for environmental variation and the logistical constraints of laboratory-based experiments, most studies use a very simplified and/or artificial social environment. However, in many group-living species, social complexity is integrated with endocrine function to regulate reproductive behavior. For example, in multi-female groups of rhesus monkeys, *Macaca mulatta*, males direct their reproductive behavior toward females only during the peri-ovulatory phase (Wallen and Winston, 1984). In pair-housing, however, males direct reproductive behavior toward females during both the follicular and peri-ovulatory phases (Wallen and Winston, 1984). Females are receptive to copulation only during particular reproductive stages in rhesus monkeys (Wallen and Winston, 1984), as well as in other vertebrates, such as rodents (Kow et al., 1978) and amphibians (Lynch et al., 2006). Thus, parameters of the mating system are shaped by cyclical ovarian patterns, which dictate the expression of specific reproductive behaviors in both males and females.

Social modulation of androgen levels is generally transient and occurs in animals during or right after an aggressive challenge. In song sparrows for example, winners of an aggressive encounter have high plasma T, while losers have low plasma T (Wingfield et al., 1990). These changes in plasma T are only seen during the breeding season, when the animals are already experiencing increased with marked increases in gonadal androgen synthesis (associated with spermatogenesis). Androgen synthesis is positively correlated with circulating androgens and sexual and territorial behavior (Borg, 1994). Song sparrows also maintain territorial aggression outside the breeding season despite low circulating androgen levels (Caldwell et al., 1984; Soma, 2006). One mechanism by which aggression during the non-breeding season can be maintained is through brain synthesis of androgens (Pradhan et al., 2010b). Thus, the behavioral output of an organism is affected by variation in both responses to social interactions and endocrine mechanisms of behavioral regulation.

Traditionally, hormone manipulations have been used to understand the role of steroids in the proximate regulation of behavior. The probability of increasing or decreasing the expression of behavior (e.g., aggressive, sexual) using a hormone manipulation is complicated because behaviors are often exhibited within tightly regulated windows that depend on the integration of ecological, social, and endogenous factors. For example, pharmacological treatment of spotted antbirds (*Hylophylax n. naevioides*) with androgen and estrogen inhibitors is during the breeding season is more effective in regulating aggressive behavior than during the non-breeding season. Effects also depend on the intensity of aggressive stimuli (Beebee, 2004). Experimentally elevated hormones often have impacts only during a short period after the manipulation and only specific components of aggression may be affected. In black redstarts (*Phoenicurus ochruros*), hormone manipulation causes short-term decreases in T and affects only specific components of song structure (Apfelbeck et al., 2013). Moreover, these effects also depend on whether the behavior occurs in a conspecific aggression or mating context (Apfelbeck et al., 2013). Thus, timing and social group dynamics are both important factors for determining the proximate hormonal mechanisms regulating behavior.

### Insights from *Lythrypnus dalli*

**Figure 7:**
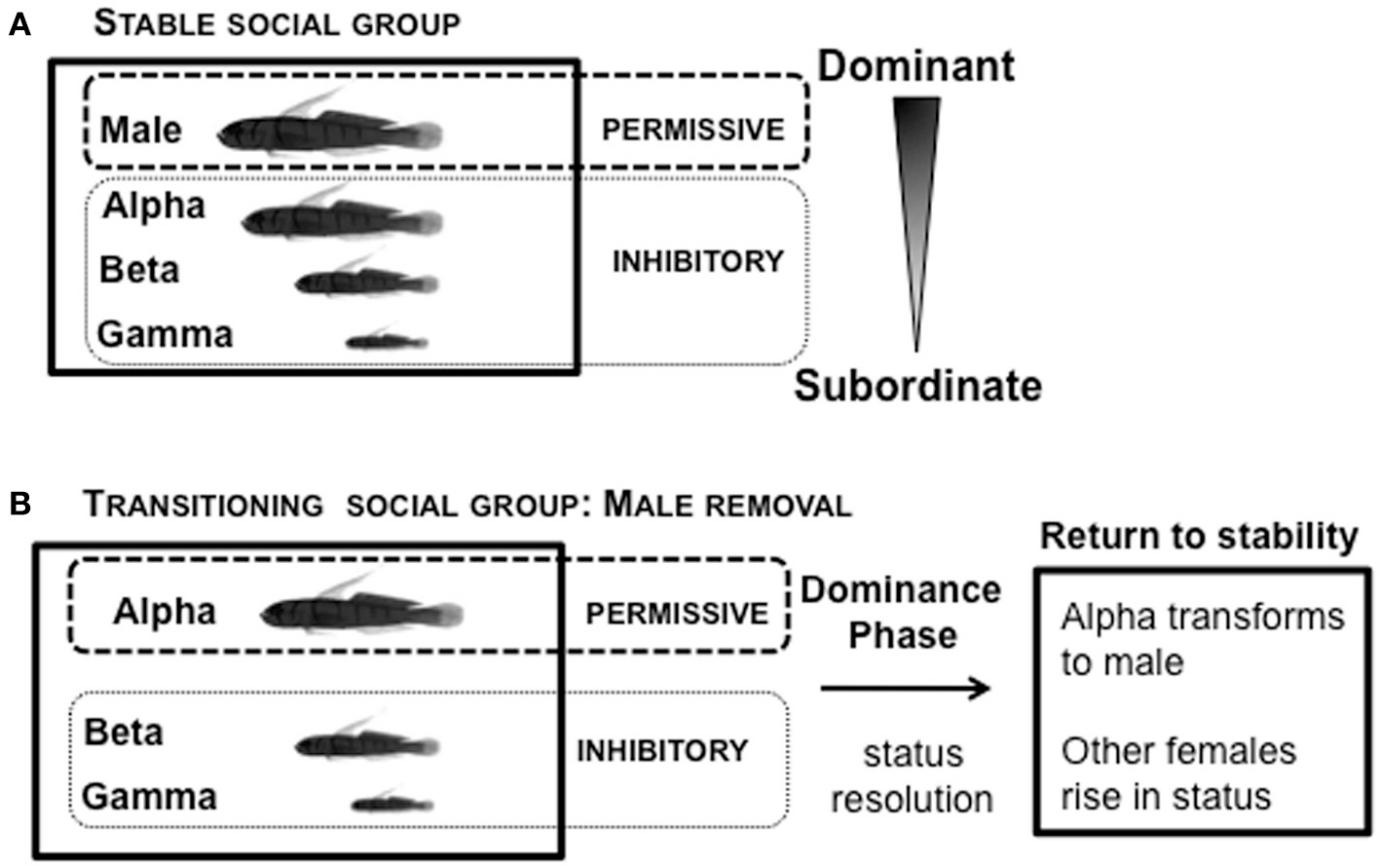
**Social context of *Lythrypnus dalli*. (A)** Stable social groups form a linear social hierarchy in the laboratory within 5 days. In the presence of the male, all females are in an environment *inhibitory* to sex change. **(B)** Sex change can then be readily induced in the alpha female by removing the male, which changes the social context. The most dominant female is now in a transitioning social environment that is *permissive* for sex change, while the subordinate females are in an environment inhibitory for sex change. Depending upon the specific group, there is substantial variation in the time over which a stable social hierarchy is re-established (“Dominance Phase”), after which there is a decline in rates of agonistic interactions.

In the laboratory, *L. dalli* social groups are easily set up under conditions permissive for natural sex change and spawning (Figure 7). The male, the most dominant member of the social group, is provided with a harem of 2–3 size-mismatched females and a piece of PVC tube that serves as his nest. These groups establish a robust linear hierarchy within 5 days (Reavis and Grober, 1999). The male regulates female entry into the nest for spawning. The presence of eggs in the nest is important for understanding endocrine state: males only show high systemic KT levels when they are actively caring for eggs in their nest, but not when eggs are absent (Rodgers et al., 2006). In a stable social group, in the presence of the male, all females are in an environment inhibitory to sex change (Figure 7A). Immediately following male removal (first few minutes), the rates of agonistic interactions among the females increase while they re-establish social status relationships (Reavis and Grober, 1999). The highest-ranking female, usually the most aggressive, is not subordinated by any other female (Rodgers et al., 2007), and rapidly assumes the dominant/male position. The most dominant female is now in a transitioning social environment that is permissive for sex change, while the subordinate females are in an environment inhibitory for sex change (Figure 7B). There is substantial variation in the time required to re-established a stable social hierarchy (“Dominance Phase”), after which rates of agonistic interaction decline (Reavis and Grober, 1999).

**Figure 8:**
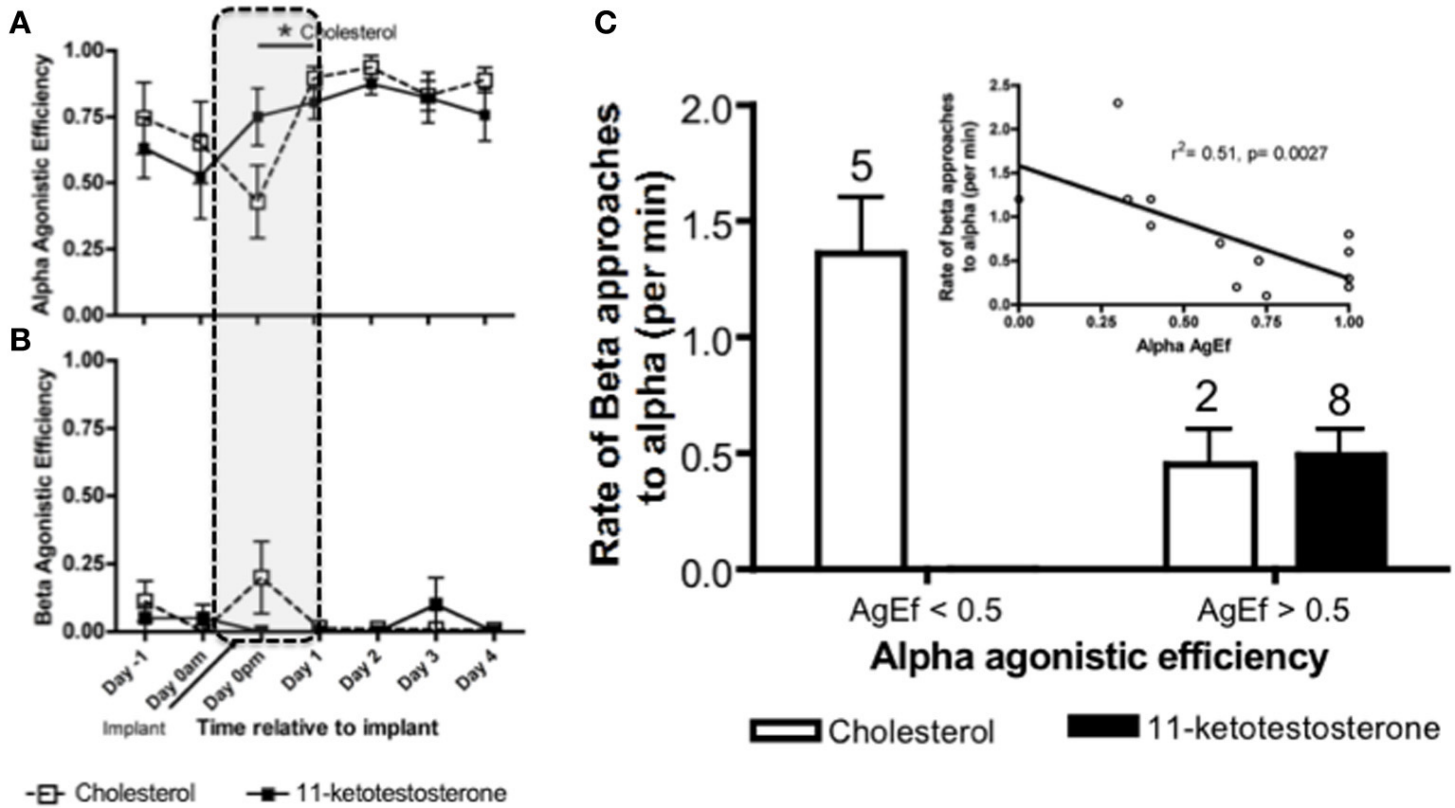
**Social context dependent effects of systemic (intraperitoneal) 11-ketotestosterone (KT) implants on agonistic interactions in *Lythrypnus dalli***. Alpha females were implanted with either KT or cholesterol and placed in a social environment that was permissive for sex change. **(A)** Alpha agonistic efficiency (AgEf) (displacements/approaches) toward betas relative to time after implant. Alpha females implanted with cholesterol had a decline in agonistic efficiency during the Dominance Phase (represented by dotted lines), but had dramatically higher agonistic efficiency the following day. **(B)** Beta Agonistic efficiency toward alphas relative to time after implant (*n* = 8 Chol and *n* = 10 KT). **(C)** Categorical representation of rates of beta approaches when alpha agonistic efficiecy was < or > 0.5. The numbers above bars represent the number of groups for each category. The inset is a regression of alpha agonistic efficiecy against rates of betas approaching alphas after alphas were treated with either KT or Cholesterol (*N* = 15). Note that some individuals were excluded from this analysis due to zero rates of interaction and overlap does not allow all data points to be seen clearly, ^*^p < 0.05.

Within 24 h of male removal, the dominant female undergoing sex change has transient increases in water-borne (Earley and Grober, unpublished results) and brain KT (Lorenzi et al., 2012). Water-borne F levels also transiently increase in the dominant female undergoing sex change and remain elevated up to 3 days, after which F declines to basal levels (Solomon-Lane et al., 2013). In pairs of size-mismatched females, implanting (IP) the subordinate (beta) female with KT causes her to transform morphologically into a male. However, because the social context predominates, betas retain their female-typical behavior and subordinate social status (Rodgers, 2007). In another experiment involving pairs of size-mismatched females, the dominant individual (alpha) undergoing sex change was implanted IP with KT or cholesterol (Figure 8). Aggressive behaviors increased transiently during the critical period of social instability but did not persist as a result of KT treatment (Pradhan et al., 2014a). Agonistic efficiency, a composite score of aggression (rate of displacements/rate of approaches) (Solomon-Lane et al., 2014), was only affected in cholesterol implanted individuals (Figure 8A). Alpha females implanted with cholesterol initially (Dominance Phase) exhibited a decline in agonistic efficiency but had dramatically higher agonistic efficiency the following day (Pradhan et al., 2014a). Overall, the data show that effects of the KT or cholesterol treatment cascade through the social group, such that in groups where alphas are less effective agonistically, betas have higher approach rates during the Dominance Phase (Pradhan et al., 2014a) (Figures 8B,C).

Transitioning *L. dalli* social groups also offer other opportunities to investigate the role of social and endocrine context on other types of behaviors. As mentioned above, in stable groups, males aggressively maintain their territory, control access to the nest, and exclusively exhibit parenting behavior. Through the use of ICV injections of CBX, we found that neural KT regulates male parenting behavior (Pradhan et al., 2014c). A new social context is created when specific ICV manipulations inhibits males from guarding their nests, allowing alpha females to enter the nesting site. Curiously, some alphas, in the absence of parenting males, demonstrate parenting behavior. After only 1 h, these parenting females that have significantly higher brain KT and lower gonadal KT compared to those alphas with males in the nest (Pradhan et al., 2014c). Levels of KT in betas are not affected, suggesting that behavioral and endocrine changes are specific to the expression of a particular behavior and not the sex of the individual. Additionally, there were no changes in T, E_2_, or F levels (Pradhan et al., 2014c). Our results demonstrate that rapid changes in social context can have differential effects on local levels of specific steroids.

### Synthesis: from broadcasting for general effect to local signaling for specific effect

We have presented a variety of endocrine and social factors that need to be integrated to elucidate how an animal expresses a phenotype. Proxies of endocrine function are based on the fact that centers in the hypothalamus and pituitary release regulatory hormones that stimulate the gonad or the adrenal gland to produce potent sex steroids or glucocorticoids, respectively. The traditional assumption follows that large quantities of steroids produced by endocrine organs, the “source,” floods the circulatory system, and hormones are then transported to specific target organs to cause downstream effects on phenotype. A corollary to this conventional view is that gonadal steroids provide a system-wide signal that advertises or “screams” to the rest of the body that the gonad is ready to release gametes. Note that the scream is not directed. It is a systemic broadcast of remarkably high intensity and is a by-product of gonadal function. This “Screaming Gonad” hypothesis is generally applicable to species whose mating behavior is heavily regulated as a series of restricted reproductive cycles within and between mating seasons. In an evolutionary sense, the gonad does not know that it is screaming to the body; however, parts of the body express receptors to “hear” the signal. This has allowed for the coordination of reproductive function across many body tissues. For example, increased T in male rodents allows for the activation of sexual behavior, while gonadectomy gradually eliminates expression of sexual behavior (Damassa et al., 1977). These data indicate that most of the circulating T in rodents is likely to be gonadally produced. Testosterone acts as a signal to coordinate the expression of reproductive traits through affects on multiple organs that, together, enhance reproductive success. Gonadal T produced during the breeding season (when rats express reproductive behavior) is generally very high; however, the quantity of T required to maintain reproductive behavior is much lower (Damassa et al., 1977), suggesting that spermatogenesis drives T to circulating levels that are much higher than is needed to change physiology and behavior. Sexual selection results in direct fitness consequences and, as such, should co-opt the hormones that regulate gamete production to exert control over the expression of sexual behavior. Evolutionarily speaking, the rest of the organism is then entrained to reproductive signals from the gonad, so that all efforts of the organism work in a coordinated fashion toward increasing reproduction and thus fitness.

The mechanisms involved in the organization and activation of sexual behavior were proposed based primarily on work in rodents, starting with the classical study by Phoenix et al. (1959). Rodent models remain prevalent in laboratories investigating the function and mechanism of hormonal action, and the emergent principles derived from rodent models can have broad application. However, there are many species that express a level of sexual plasticity that cannot be explained by this existing model, primarily because many of the characteristics of adult sexual phenotype (Phoenix et al., 1959) are organized and therefore fixed during early development. In contrast to mammals that generally exhibit sexual canalization *in utero*, there are many species that have more flexibility and avoid the early fixation of sex. In these species, sex determination is environmental, not chromosomal (Devlin and Nagahama, 2002). For species in which the social environment determines sex, activating molecules, such as steroid hormones, play an important role in the reorganization of anatomy and behavior and maintaining sexually dimorphic phenotypes. For example, males generally have higher levels of circulating androgens than females. Males of species that show unidirectional sex change, such as wrasses, generally maintain dramatically higher circulating KT levels than females (Perry and Grober, 2003), and this might serve as a mechanism to canalize the terminal male phenotype. Species that have the capacity for bi-directional sex change, such as *L. dalli*, tend not to have dramatic sex differences in circulating androgen levels (Lorenzi et al., 2008, 2012), and this may allow them to avoid canalization. It is noteworthy that the absence of sexual dimorphism in KT levels in *L dalli* is a result of very low KT levels in males rather than elevated KT levels in females. While relatively low circulating KT levels in males may provide a mechanism that allows for male to female sex reversal, it does not preclude males from using KT to regulate sexually dimorphic behavior. One mechanism that allows for this behavioral plasticity is the local regulation of steroidogenesis and steroid signaling (e.g., brain KT is necessary for *L. dalli* parenting). Local regulation is the dissociation of specific endocrine glands as control centers, and allows specific target tissues, rather than endocrine glands, to control phenotypes.

The conventional view of the endocrine system that gonadal steroids regulate all aspects of reproductive behavior, allows for variation in environmental and social context to modulate the expression of behavior. Thus, in addition to circulating steroids entraining reproductive physiology, anatomy, and behavior, we should also expect to find local brain mechanisms that allow for contextual regulation of reproductive behavior. Local, tissue-specific regulatory systems provide checkpoints for the globally dispersed signal (Brenowitz and Lent, 2002). Gonadal function in some species can be regulated by social interactions, and rapid and fine-tuned control of behavior could only occur via local modulation. We propose that the activational phenotypic effects of hormones that are beneficial for attracting mates (e.g., the biochemical cascade of events involved in increasing muscle mass or change in coloration) could be triggered initially by a gonadally generated cue. If steroidogenic machinery (enzymes, cofactors, and substrates) is present in those initial steroid targets, then local synthesis of hormones (and receptors) could maintain those phenotypic effects. Gonadally produced sex steroids might be sequestered and used by those organs that do not have the capacity for local synthesis. For example, the supracarinalis muscle of *L. dalli* males expresses both, 11β-HSD and androgen receptor, while the external genetilia express androgen receptor but not 11β-HSD (Schuppe et al., unpublished results). These data suggest that while the muscle has local steroidogenic potential and could maintain its androgenic dimorphism via local regulation, the external genetilia only have the machinery for sensing androgen and is strictly a target organ.

Dissociating endocrine glands from their roles as control centers might serve as a mechanism to maintain reproductive plasticity by allowing more local control of target tissues phenotype. In those cases, the brain can regulate the expression of behavior regardless of signals from the gonad. The emancipation from strict gonadal control of reproductive behavior has occurred in a variety of species (humans, bonobos, sex changing fish) and seems to be associated with social systems where individuals can accrue non-procreative benefits from engaging in sexual behavior (e.g., resources, resolution of social conflict, manipulation of social hierarchy). In all of these cases, release from control by the “screaming gonad” requires the engagement of specific local mechanisms for regulating the expression of reproductive traits and, specifically, the control of courtship, mating, and parenting. To reduce the possible overstimulation produced by the sex hormones released from the “screaming gonad,” and for reduction of unnecessary stimulation of target organs, specific receptors on either the cellular or nuclear membrane initiate the downstream intracellular events (Nelson, 2011). In order to maintain within-tissue control and be receptive to local steroid signals, tissues could filter out the high intensity gonadal “scream.” Thus, binding globulins might be the proteins that bind with hormones in general circulation to regulate the unnecessary activation of target organs.

There are examples from a range of vertebrates, from fish to humans, that provide support for local brain regulation of reproductive behavior. First, female wrasses can behaviorally change sex to males when the social environment is permissive, even in the absence of gonads (Godwin et al., 1996). Second, when male *L. dalli* are ICV injected with CBX, a KT synthesis inhibitor, their parenting behavior is blocked (Pradhan et al., 2014c). However, upon delivery of KT along with CBX rescues those effects (Pradhan et al., 2014c). Thus, neurally produced KT can regulate male nest care behavior, and there is little evidence for gonadal involvement in the regulation of parenting. Finally, some primates, including human females, regularly display sexual activity outside of the fertile period of their reproductive cycle. These data demonstrate the presence of local control mechanisms, facilitated in some cases via local production of hormones. According to this view, hormones that originally were produced by the gonad, are now also being synthesized in the brain so as provide rapid regulation of behavior, allowing for a more adaptive matching to immediate context. Local synthesis of steroids allows for peripheral tissues to commandeer the ancestral pathway used for steroidogenic regulation, but under novel control processes that can be more carefully regulated in both time and space. Synaptocrine signaling (Saldanha et al., 2011) provides a cellular mechanisms within brain circuits that can exercise local control of behavior.

## Overall significance and future directions

**Figure 9:**
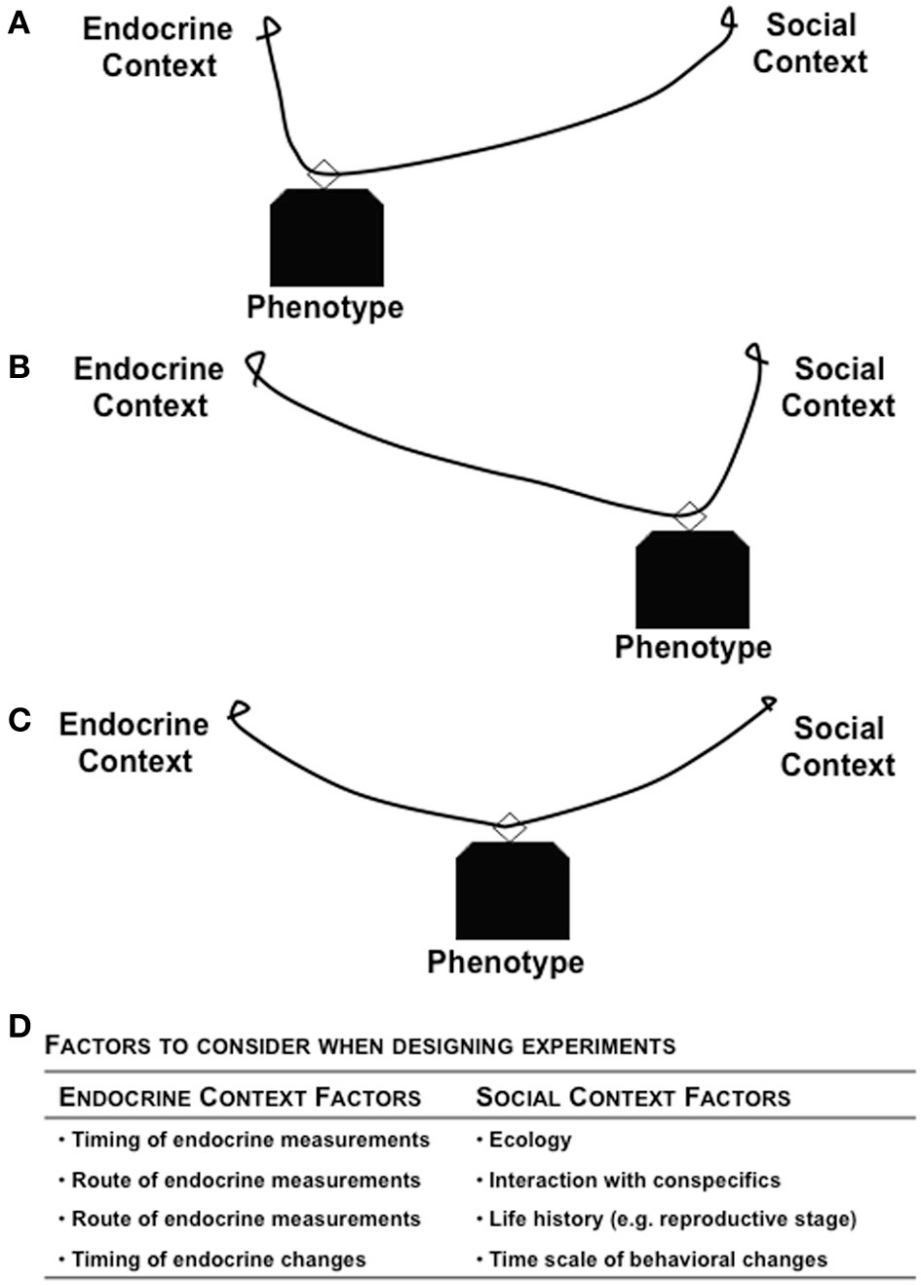
**Functional interpretation of endocrine studies should be cognizant of the relevant proxy**. Loose links exist, such that expression of phenotype is dependent upon both, **(A)** Endocrine and **(B)** Social factors. **(C)** Balance between these two factors is necessary for the regulation of function and increase lifetime reproductive success. **(D)** Several endocrine and social context factors must be considered when designing experiments.

The organization of the life cycle into distinct life history stages allows for specific adaptations to the unique environment experienced at each stage and the exploration of large and small scale mechanisms associated with the regulation of phenotypic traits. In an effort to increase fitness, individuals must respond to different social contexts in appropriate ways. The behavioral, physiological, and morphological components of these responses are closely regulated by the endocrine system. The use of validated fitness and endocrine proxies under different environmental (social and physical) contexts substantially add to our depth of knowledge regarding mechanisms that regulate the expression of context-dependent phenotypes. Caution should be exercised when interpreting results and drawing conclusions about the similarities and/or differences in mechanisms regulating phenotype based on proxies of measurement, because any two proxies may provide for widely different interpretations. Hypothesis testing should involve experimental designs that consider both the social and environmental context (Figure 9). According to our proposed model, the relative contribution of the particular social and/or endocrine context being investigated act together to affect phenotype. One or the other component may be more important in particular environments, but a balance is established, which allows for the expression of an adaptive phenotype in a variety of social and/or biotic environments. Finally, it is apparent that the comparative study of diverse species provides insights into unique mechanisms of behavioral regulation. While the establishment of small scale spatial (local steroid synthesis and action) and temporal (rapid time scale) effects of steroids have spurred a re-evaluation of the mechanisms by which hormones exert their effects, tremendous gaps in our knowledge remain. Given the diversity of mechanisms known to regulate steroid actions, a comparative species approach that integrates hormones and behavior in the service of fitness will greatly facilitate progress in this field.

### Conflict of interest statement

The authors declare that the research was conducted in the absence of any commercial or financial relationships that could be construed as a potential conflict of interest.
